# Bacterial ACC deaminase: Insights into enzymology, biochemistry, genetics, and potential role in amelioration of environmental stress in crop plants

**DOI:** 10.3389/fmicb.2023.1132770

**Published:** 2023-04-27

**Authors:** Mohammad Shahid, Udai B. Singh, Mohammad Saghir Khan, Prakash Singh, Ratan Kumar, Raj Narian Singh, Arun Kumar, Harsh V. Singh

**Affiliations:** ^1^Plant-Microbe Interaction and Rhizosphere Biology Lab, ICAR-National Bureau of Agriculturally Important Microorganisms (NBAIM), Mau, Uttar Pradesh, India; ^2^Department of Agricultural Microbiology, Faculty of Agricultural Sciences, Aligarh Muslim University, Aligarh, Uttar Pradesh, India; ^3^Department of Plant Breeding and Genetics, Veer Kunwar Singh College of Agriculture, Bihar Agricultural University, Dumraon, India; ^4^Krishi Vigyan Kendra, Rohtas, Bihar Agricultural University, Bikramganj, Bihar, India; ^5^Directorate of Extension Education, Bihar Agricultural University, Bhagalpur, Bihar, India; ^6^Swamy Keshwanand Rajasthan Agriculture University, Bikaner, Rajasthan, India

**Keywords:** environnemental stress, ethylene, plants, PGPR, ACC deaminase, mode of action

## Abstract

Growth and productivity of crop plants worldwide are often adversely affected by anthropogenic and natural stresses. Both biotic and abiotic stresses may impact future food security and sustainability; global climate change will only exacerbate the threat. Nearly all stresses induce ethylene production in plants, which is detrimental to their growth and survival when present at higher concentrations. Consequently, management of ethylene production in plants is becoming an attractive option for countering the stress hormone and its effect on crop yield and productivity. In plants, ACC (1-aminocyclopropane-1-carboxylate) serves as a precursor for ethylene production. Soil microorganisms and root-associated plant growth promoting rhizobacteria (PGPR) that possess ACC deaminase activity regulate growth and development of plants under harsh environmental conditions by limiting ethylene levels in plants; this enzyme is, therefore, often designated as a “stress modulator.” TheACC deaminase enzyme, encoded by the *AcdS* gene, is tightly controlled and regulated depending upon environmental conditions. Gene regulatory components of *AcdS* are made up of the LRP protein-coding regulatory gene and other regulatory components that are activated *via* distinct mechanisms under aerobic and anaerobic conditions. ACC deaminase-positive PGPR strains can intensively promote growth and development of crops being cultivated under abiotic stresses including salt stress, water deficit, waterlogging, temperature extremes, and presence of heavy metals, pesticides and other organic contaminants. Strategies for combating environmental stresses in plants, and improving growth by introducing the *acdS* gene into crop plants *via* bacteria, have been investigated. In the recent past, some rapid methods and cutting-edge technologies based on molecular biotechnology and omics approaches involving proteomics, transcriptomics, metagenomics, and next generation sequencing (NGS) have been proposed to reveal the variety and potential of ACC deaminase-producing PGPR that thrive under external stresses. Multiple stress-tolerant ACC deaminase-producing PGPR strains have demonstrated great promise in providing plant resistance/tolerance to various stressors and, therefore, it could be advantageous over other soil/plant microbiome that can flourish under stressed environments.

## Introduction

Plant growth and productivity are affected by myriad complex factors, both physiological and environmental, including plant genotype, soil physical and chemical characteristics, availability of nutrients, and other variables (Schwachtje et al., 2019). In addition, crop growth and yield may be stressed by several biotic and abiotic factors, i.e., salinity, drought, temperature, mechanical wounding, waterlogging, organic contaminants, heavy metals and other xenobiotics (Gupta et al., 2013; Gull et al., 2019). As a consequence of these factors, ~35–50% yield loss has beed reported so far in major crops globally (Stallworth et al., 2020). Abiotic stresses are therefore considered as a primary influence affecting agricultural production worldwide.

Global food supplies must be increased to fulfil the increasing demands of rapidly-growing populations (Place et al., 2017). Response to several biotic and nutritional challenges in plant husbandry can be resolved using chemical pesticides, fertilizers, and other agrochemicals. However, using non-biological methods to address problems posed by abiotic stressershas its share of difficulties. Plants respond to external challenges by altering production of certain hormones, which promotes the synthesis of stress-related proteins that afford protection against the negative effects of stressors (Gupta et al., 2020). In this regard, ethylene is considered as the most common phytohormone mediating stress response in many crop plants (Tiwari et al., 2020). In contrast, when ethylene production exceeds a certain threshold, it becomes “stress ethylene.” Excessive levels of ethylene adversely affect proliferation of roots, shoots, and other yield parameters and, thus, hamper overall plant performance (Klay et al., 2018; Mog et al., 2018). The detrimental impacts of the high ethylene levels can be reduced by various soil/plant-colonizing microbiomes that contain the essential enzyme ACC deaminase (Glick, 2014; Saikia et al., 2018). ACC deaminase (ACCD) converts the harmful form of ethylene to a non-toxic state (Das and Osborne, 2018). The ACCD decreases ethylene levels in plants by breaking down ACC into α-ketobutyrate (C_4_H_6_O_3_) and ammonia (Bharti and Barnawal, 2019) which in turn allow roots/shoots or entire plants to grow normally (Glick, 2014). Thus, ACCD permits plants to thrive in challenging environments by reducing harmful concentrations of ethylene (Han et al., 2015; Ravanbakhsh et al., 2017; Sarkar et al., 2018a,b). ACC serves as the originator of ethylene in plants (Ouaked et al., 2003). “Induced systemic tolerance” refers to the inherent characteristics of assigning tolerance to abiotic stressors through ACCD activity and some redundant PGPR processes to alleviate stresses in host plants (Arya et al., 2018; Carlson et al., 2020). Therefore, PGPR equipped with ACCD activity are essential organisms that play a major role in the reduction/mitigation of the toxic effects of several environmental stressors such as salinity, drought, heavy metals, and organic pollutants (Table 1). The production of the stress hormone, ethylene, and its impact on plants while growing under stress has previously been explained. Taking relevant papers into account, the present review describes the importance of ethylene in plant physiology and the function of bacterial ACC deaminase in reducing stress-induced ethylene levels in plants, thereby circumventing the negative effects of environmental stressors.

**Table 1:** Examples of ACC deaminase producing PGPR.

PGPR strains	Source	ACC deaminase activity (nmol α-ketobutyrate mg protein^−1^ h^−1^)	Reference
*Achromobacter xylosooxidans* A551	*Pisum sativum* rhizosphere	400 ± 4.0	Belimov et al. (2009)
*Burkholderia cepacia* PSBB1	*Vicia faba* rhizosphere	–	Shahid and Khan (2018)
*Pseudomonas putida* UW4	–	3,030 ± 60	Hontzeas et al. (2006)
*Rhizobium leguminosarum* 128C53K	*Pisum sativum* rhizosphere	5.0 ± 1.0	Belimov et al. (2005)
*Serratia proteamaculans*	Rhizosphere region of salt-affected *T. aestivum* (L.)	276 ± 00	Zahir et al. (2009)
*Serratia marcescens* BC-3	Rhizosphere soils of salt and petroleum amended *E. crusgali* plants	38,520 ± 00	Liu et al. (2015)
*Bacillus pumilus* SB1-ACC3	*Oryza sativa* (L.) rhizosphere	1,460 ± 00	Bal et al. (2013)
*Bacillus licheniformis* B2r	Salinity-stressed rhizosphere soils	860 ± 00	Chookietwattana and Maneewan (2012)
*Bacillus* sp. MR4	Rhizosphere soils of *Arabidopsis thaliana* (L.) and *Festuca rubra* (L.)	15,920 ± 00	Grobelak et al. (2018)
*Bacillus cereus* LB1	Tissue of *Carthamus tinctorius* (L.)	2,400 ± 00	Hemida and Reyad (2018)
*Bacillus aerius* SB1	Tissue of *Carthamus tinctorius* (L.)	1,800 ± 00	Hemida and Reyad (2018)
*Pseudomonas* sp. R3	Rhizosphere soil of *Arabidopsis thaliana* (L.) and *Festuca rubra* (L.)	23,490 ± 00	Grobelak et al. (2018)
*Mesorhizobium ciceri* strain LMS-1 (pRKACC)	–	2,305 ± 00	Nascimento et al. (2012)
*Sinorhizobium meliloti* KYA71 and KYA40	Soil and Water Research Institute (Iran)	326,136 ± 00	Khosravi et al. (2014)
*Serratia ficaria*	Salinity-stressed rhizosphere soils	326 ± 00	Nadeem et al. (2010)
*Pseudomonas* sp. ST3	Rhizosphere soil of *Ipomoea aquatica* (L.)	900 ± 00	Trung et al. (2016)
*Enterobacter aerogenes*	Rhizosphere soil of salt-treated *Zea mays* (L.)	341 ± 00	Nadeem et al. (2007)
*Enterobacter* sp. CS1	-	170 ± 00	Huang et al. (2013)
*Enterobacter cloacae* ZNP-4	*Ziziphus nullifera* (L.) rhizosphere soil	188.90 ± 9.3	Singh et al. (2022)
*Pseudomonas* sp. TR15a	Contaminated rhizosphere of *Trifoliumrepens* (L.)	53.74 ± 00	Kumar et al. (2021)
*Bacillus amyloliquefaciens*	Pearl millet rhizosphere	2196.23 ± 00	Murali et al. (2021)
*Ensifer adhaerens* KS23	Rhizosphere soil of leguminous crop	174.2 ± 00	Katiyar et al. (2021)
*Achromobacter* sp.	Rhizosphere soil	4.90 ± 00	Sun et al. (2022)
*Streptomyces hydrogenans* DH16		363 ± 00	Kaur and Manhas (2022)

### Ethylene: Biosynthesis, physiology, regulation, and stress response in plants

**Figure 1:**
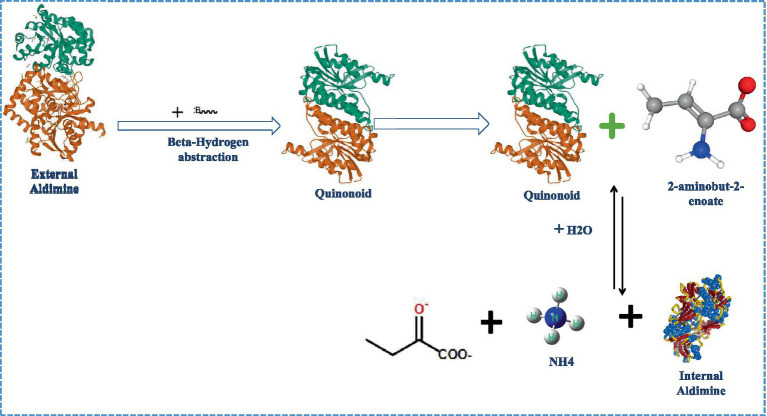
Elucidation of route1 (Direct β-hydrogen extraction) for ACC metabolism by ACC deaminase.

Ethylene, the smallest and simplest gaseous phytohormone produced by plants, regulates a suite of biological and functional processes in plants (Light et al., 2016; Fernandez-Moreno and Stepanova, 2019). Processes regulated by ethylene include seedling germination, ripening/maturation of fruit, senescence, development of root hairs and nodules, elongation of roots, and epinasty (Zhang et al., 2016; Zhu et al., 2016; Sun et al., 2019; Figure 1).

Ethylene production in plants is primarily influenced by environmental factors and depends on the degree and intensity of environmental variables. The identification of ethylene as a plant growth regulator was revealed by early leaf shedding, geotropism of etiolated pea seedlings when exposed to lighting gas, and the ripening/maturation of plant organs when exposed to kerosene combustion gas (Pierik et al., 2006; Glick et al., 2007a).

A wide array of biotic and abiotic factors (e.g., salinity, drought, waterlogging, flooding, agrochemicals, pesticides, heavy metals, organic and inorganic pollutants, phytopathogens) inducesethyleneproduction in plants (Gontia-Mishra et al., 2014). Henceforth, the ethylene produced under such environmental stresses is regarded as “stress ethylene” (Glick, 2014). The stress ethylene triggers genes to be transcribed and further expressed, resulting in plant senescence. Ethylene biosynthesis in plants follows a relatively straightforward system where methionine is converted to S-adenosyl methionine (SAM) by the enzyme SAM synthetase that is subsequently used as a substrate by ACC synthase to generate 1-aminocyclopropane-1-carboxylic acid (ACC). The ACC generated in this process acts as precursor for ethylene production by the action of enzyme ACC oxidase.

### ACCD: Biochemical properties and mode of action

When ACC deaminase was identified in soil microorganisms for the first time, it was demonstrated to transform ACC to ammonia (NH_3_) and α-ketobutyrate, which were subsequently metabolized by microbes (Honma and Shimomura, 1978). ACCD is a pyridoxal PO₄^3−^-dependent enzyme. In order to activate the enzyme, about 3.0 mol of pyridoxal PO₄^3−^ (enzyme bound) mol^−1^ of enzyme or 1.0 mol trimeric^−1^subunitis required (Honma, 1985; Karthikeyan et al., 2004). This enzyme was first purified from *Pseudomonas* sp. strain ACP; however, strains of *P. chloroaphis* 6G5 (Klee et al., 1991) and *P. putida* GR12-2 (Jacobson et al., 1994) have also been utilized for partial purification of ACCD. The molecular mass and shape of enzyme isolated from all three sources appear to be identical. *Pseudomonas* sp. strain ACP was found to have a native size of 110–112 KDa, while *P. putida* GR12-2 had a native size of 105 KDa. In nature, this enzyme is found in the trimeric form with ~36,500 Da mass subunit.

At pH 6.0 and pH 9.0, the absorption maxima of pure ACC deaminase from *Pseudomonas* sp. were 416 and 326 nm, respectively (Honma, 1985). The 326 nm band observed at pH 9.0 could represent the activation form of ACCD to which inhibitors and substrates strongly bind (Jacobson et al., 1994). The published range of K_m_ values for enzyme extracts from various bacteria at pH 8.5 is 1.5–17.4 mM, indicating that the enzyme has a low affinity for ACC. Following second-order kinetics, the total efficiency (k_cat_/_km_) of ACC deaminase is around 690 M^−1^S^−1^. The ACC deaminase K_m_ value for 1-amino cyclopropane 1-carboxylate has been established using enzyme extracts from microorganisms at pH 8.5 (Klee et al., 1991). Several bacterial species produced ACCD enzyme and their activity was evaluated over a broad pH range and at pH 8.0 to 8.5 showing highest activity. The optimal temperature for ACC deaminase activity is 30°C (Glick et al., 1998).

Because ACC deaminase is an inducible enzyme, its production is triggered when its substrate, ACC, is present. In *P. putida* strain GR12-2 and *Pseudomonas* sp. strain ACP GR12-2, the lowest level of substrate for induction was determined to be 100 nM. ACCD induction is a lengthy and complex procedure. Within a few hours of incubation with the substrate, the enzyme expresses its activity which, steadily declines thereafter (Jha et al., 2012). In a minimal medium supplied with (NH₄)₂SO₄ (ammonium sulfate) as Nsource, the basal level of enzyme activity was observed. It was further demonstrated that growing bacteria in a minimal medium that contained ACC as the only N source led to increased enzyme activity, suggesting that the substrate ACC had a direct relationship with induction of enzyme activity (Honma, 1983). Expression of ACCD and the activation of other amino acids such as *L*-alanine, *DL*-alanine, and *D*-serine, increase to a lesser degree than in the case of ACC. Furthermore, both ACC and amino-isobutyric acid (C_4_H_9_NO_2_) produced a similar degree of enzyme activity in *Pseudomonas* sp. strain ACP (Honma, 1983). According to Glick et al. (1998), ACC is released from plant roots or seeds, ingested by soil microbiota, and hydrolysed to ammonia and α-ketobutyrate. The quantum of ACC outside the plant root, however, decreases due to ACC absorption and hydrolysis. The equilibrium between levels of internal and external ACC is also maintained by the exudation of excess ACC into the rhizosphere. As a result, a reduction in ACC levels reduces the production of stress hormone ethylene in host plants and stimulating growth of the plant (Glick et al., 1998).

*L*-isomers of amino acids such as *L*-alanine, *L*-serine, *L*-homoserine, and *L*-aminobutyric acid inhibit ACC deaminase competitively, with *L*-alanine and *L*-serine exhibiting greatest inhibition. ACC deaminase isolated from *Pseudomonas* sp. can also use ACC-related compounds like 2-alkyl-ACC and vinyl-ACC as substrates. Strain ACP, although the enzyme has a peculiar preference for *D*-amino acids, being inactive with any *L*-amino acids or derivatives. According to NMR research, a proton is removed from the β-carbon of *D*-alanine but not from the *L*-isomer. These findings support the stero-specific breakage of the cyclopropane ring during ACC deamination, which explains the deamination of *D*-amino acids and many substituted *D*-alanines. The iodoacetamide derivative 1,5 N-iodoacetamidoethyl-1-aminonapthalene-5-sulfonic acid (1,5-I-AEDANS) inactivates ACC deaminase more effectively in the presence of *D*-alanine than iodoacetamide. During inactivation, a thiol group in cysteine residue 162 is altered, as it is the aldimine connection between pyridoxal phosphate and lysine residue 51 (Honma et al., 1993). The primary feature of the ACC deaminase-catalyzed process is the opening of the ACC cyclopropane ring. The most likely method for cleaving the cyclopropane bond appear to be nucleophilic addition and elimination, although the full reaction mechanism is unknown (Thibodeaux and Liu, 2011).

### Enzymology of ACC deaminase

The deamination of ACC, the precursor of the gaseous phytohormone ethylene, is carried out by the tryptophan synthase beta (β) superfamily enzyme ACC deaminase (EC 3.5.99.7), which is dependent on the pyridoxal 5′-phosphate (PLP) molecule. To initiate the ACC deaminase enzyme activity, 1 mol pyridoxal phosphate (vitamin B6) works as a firmly bound cofactor (Singh et al., 2015). It is found in the cytoplasm of bacterial cells and has a molecular mass of 35–42 kDa (Gamalero and Glick, 2015). PLP is thought to be an inducible enzyme that requires a substrate, ACC, at a concentration of <100 nM to activate the process. By switching ACC deaminase-producing bacterial strains from nutrient-rich growth media to minimal media containing ACC as its sole N source, the induction of enzymatic activity by substrate, ACC, is proven. Other amino acids such as *D*-alanine, *L*-alanine, *D*-valine, 2-alkyl-ACC, vinyl-ACC, and 2-aminoisobutyric acid, all of which are similar to ACC in structure and behavior, can also activate ACC deaminase. Furthermore, 2-aminoisobutyric acid has the same ability to stimulate activity as ACC (Malerba et al., 1996).

Activation of ACCD has been observed at various pH levels. The pH range 8.5–9.0 has, however, been found to impart the highest efficiency for the substrate and competing inhibitors. The *L*-amino acids or their derivatives decrease the activation of ACC deaminase. At pH 9.0, the ACC deaminase absorption spectra showed the strongest band at 326 nm. The activity of *Pseudomonas putida* strain GR12-2 ACC deaminase was reported to be highest at 30°C (Jacobson et al., 1994). At pH 8.5, enzyme K_m_ value ranged from 1.5 to ~17.4 mM, indicating that it does not have a strong affinity for ACC (Hontzeas et al., 2004). The enzyme has a catalytic efficiency of roughly 690 M^−1^ S^−1^ (k_cat/km_) (Klee et al., 1994). Because ACC oxidase has a stronger affinity for ACC than ACC deaminase, the lower K_m_values indicate that ACC deaminase should be present in higher concentrations (100–1,000 fold) in order to utilize the ACC substrate before ACC oxidase and hence reduce ethylene levels (Glick et al., 1998).

### Mechanism of ACC deaminase enzymatic reaction

Stressed plants generate ACC, which is hydrolyzed by the microbial enzyme ACC deaminase to α-ketobutyrateand ammonia, thus reducing stress-induced ethylene and related growth inhibition. The elimination reaction and addition of nucleophiles that breaks the cyclopropane ring is the fundamental feature of the ACC deaminase-catalyzed second-order process (Glick et al., 2007a,b). Two possible mechanisms by which ACC deaminase carried out the deamination of its substrate ACC (Walsh et al., 1981; Zhao et al., 2003) include: (i) Direct β-hydrogen extraction in whichLys-mediated hydrolytic reactions break the cyclopropane ring when a hydrogen atom is extracted from the ACC substrate (Figure 1); and (ii) Nucleophilic addition followed by β-hydrogen extraction where ACCcarbon is attacked nucleophilically, and the cyclopropane ring is opened *via Lys*51-mediated hydrogen abstraction. The internal aldimine (imine analogue of aldehyde group) is located between the ACC deaminase lysine residue and pyridoxal phosphate cofactor. The trans-aldimination process occurs when the ACC amino group displaces the *L*-lysine residue from the enzyme active site, leading to the production of external aldimine *via* an aminyl intermediate that is present in both proposed pathways (Hontzeas et al., 2006). In route 1, a Lys basic residue on an external aldimine removes the methylene proton directly, forming quinonoid, which results in the formation of a new quinonoid molecule by protonation and electronic configuration (Joshi et al., 2012). The process continues with quinonoid nucleophilic attack by basic lysine amino residues, yielding another quinonoid and 2-aminobut-2-enoate, which is then reversibly hydrolyzed to provide 2-oxobutanoate and an ammonium ion, restoring the internal aldimine (Ose et al., 2003). Following the formation of external aldimine, route 2 departs from route 1 by performing a nucleophilic attack on the proton of the β-carbon of ACC (pro-S), resulting in the synthesis of quinonoid, followed by hydrogen removal from the carbon of ACC (pro-R). Following quinonoid production, the steps are identical to route 1 (Ose et al., 2003).

### The ACC deaminase gene and its expression

#### ACC deaminase gene

As previously mentioned, the *AcdS* gene encoding ACC deaminase has been identified in various bacterial and fungal species. ACC deaminase has recently been discovered in a variety of Gram-negative bacteria (Gontia-Mishra et al., 2017), fungi (Rauf et al., 2021), endophytes (Sofy et al., 2021) and rhizobia (Saghafi et al., 2019). An ACC deaminase gene has been identified in several species, notably *R. leguminosarum* bv. *Trifoli* and *Mesorhizobium loti* MAFF303099. The degree of ACC deaminase expression, however, differs from one organism to another. A portion of the *AcdS* gene was amplified and examined in a variety of environmental isolates using a universal pair of primers. Various workers have developed several pairs of primers to identify the presence of the bacterial *AcdS* gene. Only a few bacterial species have had the entire genetic makeup and function of the ACCD gene described (Duan et al., 2013). It has also been discovered that the nucleotide sequences of the *AcdS* gene are very similar to those of two other genes, i.e., *dcyD* and *yedO*, which encode for another PLP-dependent enzyme, D-cysteine sulfhydralase. Earlier studies have shown that certain genes previously thought to code for ACC deaminase activity also code for D-cysteine desulfhydrase (Riemenschneider et al., 2005). Nascimento et al. (2014) used *Pseudomonas* sp. strain UW-4 as a reference to evaluate the key protein residues recognized to be crucial for ACC deaminase function, including *Leu*322, *Glu*296, *Ser78*, *Tyr*295 and *Lys*51. Any alteration in residues at certain sites was considered to indicate D-cysteine desulfhydrase.

With few exceptions, the *AcdS* gene in the majority of bacterial species is chromosomal DNA-borne. In symbiotic bacteria *M. loti* (symbiont of *lotus* spp.), the ACC deaminase gene is associated with nitrogen fixation genes and might be regulated by *NifA*, which is known to activate *nif* gene expression in association with the product of *rpoN* gene (Ma et al., 2003a). Only a small fraction of the putative *AcdS* gene has been shown to encode active enzyme (Glick et al., 2013).

#### Regulation of ACC deaminase

**Figure 2:**
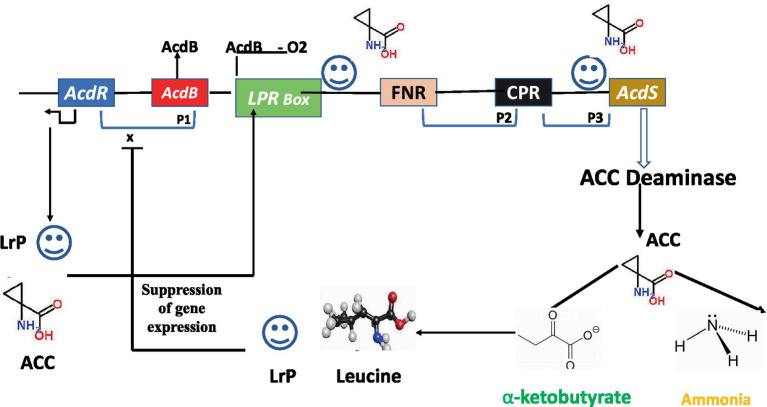
Regulatory circuits of *AcdS* gene expression in *Pseudomonas putida* UW4 and related bacteria. *AcdR*, regulatory gene for ACC deaminase; *AcdB*, encoding for glycerophosphoryl diester phosphodiester; LRP, leucine responsive protein; FNR, fumarate nitrate reductase protein; CRP, c-AMP receptor protein; *AcdS*, gene for encoding ACC deaminase.

*AcdS* is highly controlled, whose expression varies with O_2_level, quantity of substrate, and product accumulation. With few exceptions, regulation of the *AcdS*gene in various bacterial taxa is poorly understood. Li et al. (2000) presented the model for regulating ACC deaminase genes in *P. putida* strain UW-4. Regulatory elements for the expression of ACC deaminase gene consist of regulatory gene *AcdR* located 5′ upstream of ACC deaminase structural gene (*AcdS*), promoter regions for binding of regulatory proteins like Lrp box for binding of Lrp protein*, AcdB* box for binding regulatory protein *AcdB*, FNR box for binding of fumarate and nitrate reductase protein, and CRP box for binding of cAMP receptor protein (Figure 2). The LRP creates an active octamer in the presence of ACC, which binds to an ACC-*AcdB*complex (Gupta and Pandey, 2019). Glycerophosphoryl diester phosphodiesterase is encoded by the gene dB, which forms a complex with ACC. By attaching to the promoter region of *AcdS*, this triparental complex promotes its transcription. In other bacteria studied for *AcdS* gene expression, *AcdB*has not been demonstrated to play a function. Leucine, which is generated from α-ketobutyrate, a breakdown product of the ACCD-catalyzed process, inhibits expression of the ACC deaminase gene. As the quantity of leucine rises, it favors creation of inactive LRP dimers, which prevents the *AcdS* gene from being transcribed (Figure 2).

The regulatory mechanism that controls *AcdS* expression differs from bacterial species to species. The majority of bacteria have *AcdR* encoding LRP or related sequences, according to results of the IMG database analysis. LRP-like protein and the 70 promoters are also implicated in the regulation of the *AcdS* gene in *Bradyrhizobium japonicum* USDA 110 and *Rhizobium leguminosarum* bv. *Viciae* 128 C53K (Kaneko et al., 2002; Ma et al., 2003a). According to the evolutionary analysis of the *AcdS* and *AcdR*gene evolved in a similar fashion. Instead of the *AcdR* gene, *Burkholderia* sp. CCGE 1002 and *B. phymatum* STM 815 have two copies (megaplasmid and the other on the second chromosome) of the *AcdS* gene. In smaller replicons, these shards of evidence point to chromosomal rearrangement or gene insertion events. Some bacteria, such as *Achromobacterxylosooxidans* A-551 and *Variovoraxparrdoxus* 5C2, lack all the regulatory components as observed in the model bacterium *P. putida* UW4.In *M. loti*, the upstream elements of *AcdS* and *nifH* contain *nifA1* and *nifA2* (regulatory N_2_ fixing unit) and σ^54^ RNA polymerase sigma recognition site. It was hypothesized that expression of ACC deaminase in *M. loti* required the symbiotic nitrogen fixing regulatory gene *nifA*2 (Nukui et al., 2006).

**Figure 3:**
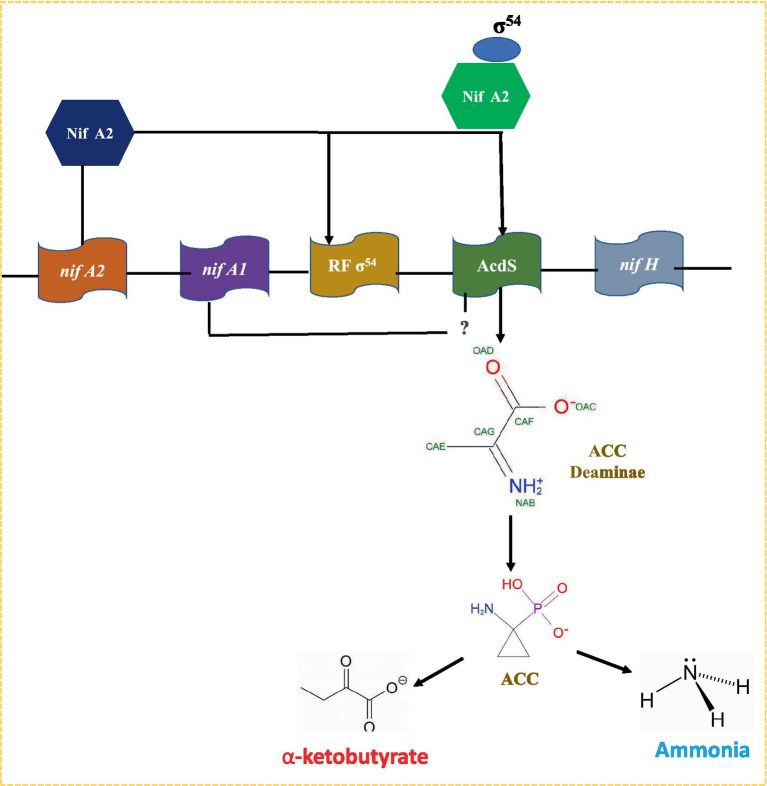
A model for *acds* gene regulation in nitrogen fixing *Mesorhizobium* sp. Expression of *acds* is positively regulated by NIFA_2_ protein which binds to σ^54^ and switch on transcription of *AcdS* gene. Nifa_1_ is also required in regulation of *AcdS* but its role is not well-understood.

The *nifA2* encoded protein *NifA2* interacts with σ^54^ RNA polymerase, favoring *AcdS* transcription. The *nifA1* also affects transcription of the *AcdS* gene to some extent; however, its role in expression of *AcdS* is not fully understood (Nukui et al., 2006; Figure 3). The *AcdS* gene is expressed in root nodules, which minimizes the negative effects of ethylene-induced senescence and increases the concentration of fixed nitrogen in nodules. The activity of ACC deaminase is commonly measured in free-living organisms; however, in *M. loti*, it was only found in symbiotic nodules (Uchiumi et al., 2004).

It must be emphasized that, unlike free-living bacteria, ACC deaminase among nodule-forming rhizobia does not reduce ethylene levels throughout the plant and, hence, cannot be employed to protect plants from various stresses (Ferguson and Mathesius, 2014; Vargas et al., 2017). Furthermore, the amount of ACCD produced in the nodule is only 2–10% of the amount produced by free-living bacteria.

The *GntR* protein coding gene is presentadjacent to the*AcdS* gene in various *Meiothermus* and *Actinobacteria*. This suggests that some downstream components may be involved in ACC deaminase expression control as well. The lack of a promoter region in some members of these genera clearly suggests that control of *AcdS* gene transcription is mediated by the interaction of the *AcdS*gene with a downstream element close to thatgene. *Brenneria* sp. EniD312, *Burkholderiaxenovorans* LB4000, and *Pantoea* sp. are examples of *Actinobacteria* and *Proteobacteria*. At-9B, a transcription regulatory element belonging to the *LysR* family was identified near the *AcdS* gene. However, it is still not clear howACC deaminase specifically functions in such organisms. Therefore, to fully comprehend the mechanism of ACC deaminase regulation and function in various bacterial genera, additional genetic and biochemical research is required.

When triggered by ACC, the putative ACC deaminase gene in *M. loti* MAFF303099 contains no regulatory elements and shows no enzyme activity (Ma et al., 2003b). ACC concentrations as low as 1 M promote ACC deaminase expression in *R. leguminosarum* bv. *Viciae* 128C53K. The introduction of the ACC deaminase and its regulatory gene from *R. leguminosarum* bv*. Viciae* 128C53K to a *S. meliloti* strain resulted in an increase in *Medicago sativa* nodulation efficiency (Ma et al., 2004). Furthermore, in terms of nodulation, the latter strain outperformed the wild type (Ma et al., 2004).

#### ACC deaminase producing PGPR: Ecological significance

**Figure 4:**
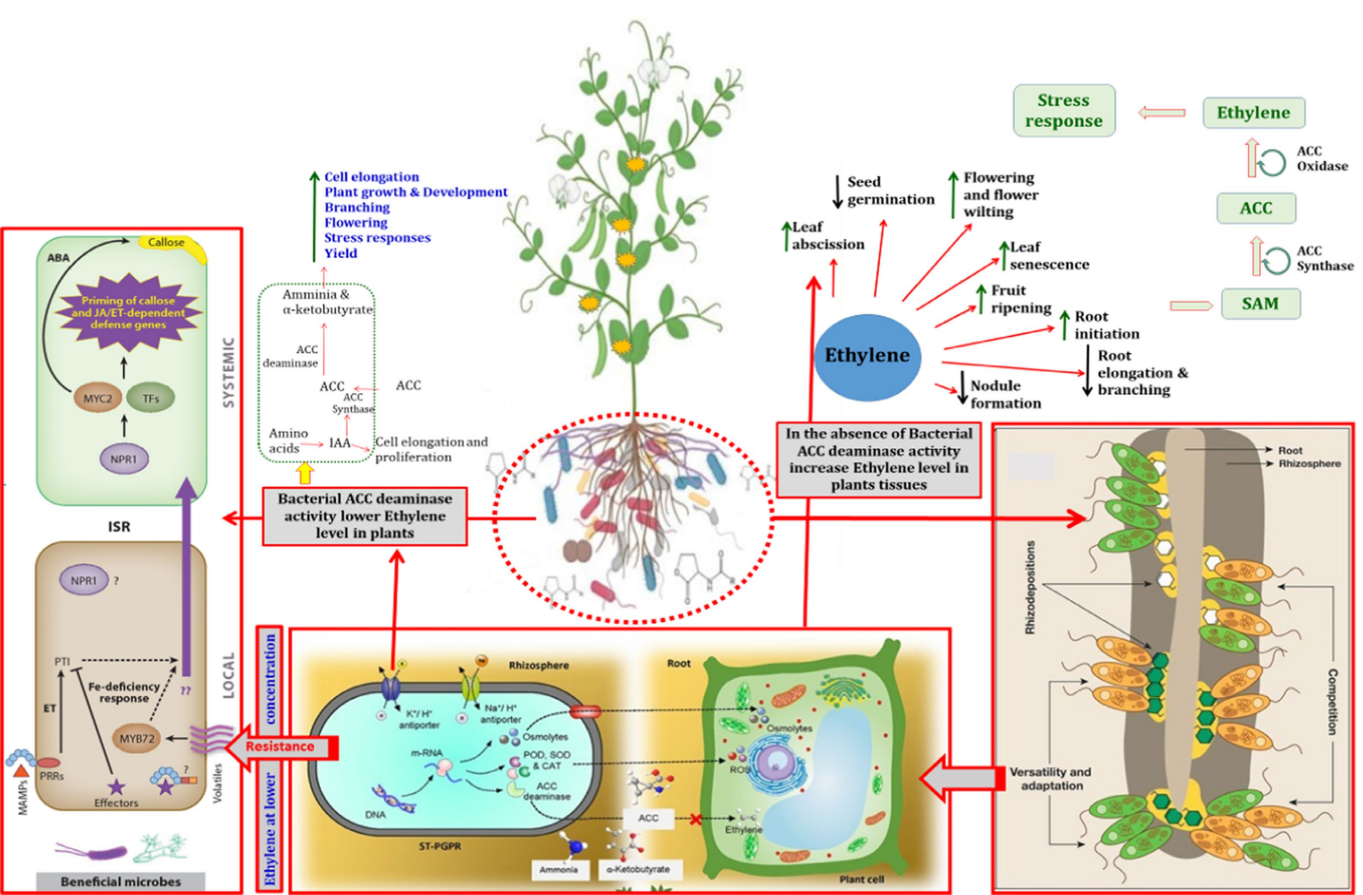
Representation of the direct and indirect roles of bacterial ACC deaminase in plant growth and development. MAMPs represent microbe-associated molecular patterns; ET, ethylene; PTI, PAMP triggered immunity; ISR, induced systemic resistance; TFs, transcription factors; ABA, abscisic acid; POD, peroxidase; SOD, superoxide dismutase; CAT, catalase; PGPRs, plant growth promoting rhizobacteria; ROS, reactive oxygen species; JA: jasmonic acid.

The relevance of PGPR having ACCD activity in reducing the effects of stress ethylene has been extensively studied. When ACCD-producing bacteria are present on the root surface of a stressed plant, they function as ACC reservoirs, reducing ethylene levels in the plant and promoting root development. Because of their extensive root growth, plants inoculated with ACCD harboring PGPR may have better tolerance to a variety of environmental challenges. Several environmental stresses (salinity, flooding, extreme temperatures, heavy metal toxicity, water deficit, nutrient deficiency, and pathogenicity) are the key limiting factors for agricultural production and productivity across the globe. It is presumed that global climate change might augment the occurrence and magnitude of environmental stresses, i.e., abiotic and biotic in the near future (Saleem et al., 2007; Timmusk et al., 2011). These stresses cause significant reduction in the crop growth and yield of stressed plants. It is well established that ethylene production increased significantly under environmental stressed condition especially in stress-sensitive crop varieties. This is commonly known as “stress ethylene” produced as a consequence of abiotic and biotic stresses. On the other hand it is well known that the ACC deaminase-producing organisms were much abundant in the rhizosphere of wild barley (*Hordeum spontaneum*) growing in a stressed environment than they were in a similar (nearby) less stressed environment (Timmusk et al., 2011). Under stresses conditions, rhizospheric and endophytic bacterial/microorganisms produces ACC deaminase which break the ACC (prerequisite of ethylene production) to α-ketobutyrate and ammonia and thereby diminishes level of “stresses ethylene” in the stressed host plants. Few reports indicated that *Methylobacterium* spp. (phytopathogenic in nature) modulate plant growth and development by decreasing environmental stress, immobilizing heavy metals, degrading toxic organic compounds and even inhibiting plant pathogens (Reinhold-Hurek and Hurek, 2011; Brader et al., 2014; Santoyo et al., 2016; Shahzad et al., 2017; Ek-Ramos et al., 2019). A number of bacteria have been discovered in soil/rhizosphere that can utilize ACC as a sole source of nitrogen, are capable of alleviating different environmental stresses, and can support improved growth and overall performance of agricultural crops (Chauhan et al., 2017; Figure 4). Plant synthesis of ethylene is also regarded as a stress response, and is closely linked to a variety of stress factors including as waterlogging, salinity, presence of heavy metals, and nutrient deficiencies (Dimkpa et al., 2009). It may be possible to apply phytoremediation at contaminated sites by taking advantage of the variation in ACC deaminase activity among microbial species under extreme environmental conditions (Glick, 2005). By biotransforming toxic substances, rhizodegradation mediated by root exudates, and/or detoxification of heavy metals, ACC deaminase-producing bacteria support plants in phytoremediation and enable host plants to thrive under challenging conditions (Qin et al., 2014). By expanding the plant root system and improving root access to soil, ACC deaminase rhizospheric bacterial populations can accelerate rhizo-remediation (Kalsoom et al., 2022). With modified root structure and architecture, inorganic pollutants are more effectively absorbed by the plant. According to Belimov et al. (2005), increased root growth was positively correlated with increased bacterial ACC deaminase activity when cadmium accumulated in plant tissue. Synthesis of minimal quantities of ethylene in leguminous plants has been shown to disrupt the *Nod* factor involved in the signal transduction pathway, which was prevented by rhizobial inoculation (Guinel, 2015). As a result, PGPR-produced ACC deaminase shields plants from the detrimental effects of ethylene when exposed to abiotic stress (Sapre et al., 2018a,b). Some widely acclaimed bacterial genera synthesizing ACC deaminase include *Achromobacter* (Sun et al., 2022), *Brevibacterium linens* (Choi et al., 2022), *Bacillus amyloliquefaciens* (Murali et al., 2021), *Ensiferadhaerens* (Katiyar et al., 2021), *Variovorax* sp. (Bessadok et al., 2020), *Enterobacter* sp. (Sagar et al., 2020), *Rhizobium* (Saghafi et al., 2019), *Bradyrhizobium* (Greetatorn et al., 2019), *Pseudomonas* (Nascimento et al., 2019), *Bacillus* (Din et al., 2019), *Burkholderia*, *Enterobacter*, *Serratia* (Zafar-ul-Hye et al., 2019), *Azotobacter* (Rizvi and Khan, 2018), *Achromobacter* (Shahid et al., 2019a,b), and *Acinetobacter*, *Alcaligenes* (Gontia-Mishra et al., 2017). Table 1 lists certain PGPR-containing ACC deaminase activity (ACCD) positive bacteria.

#### Biochemistry of ACC deaminase

ACC deaminase is a multimeric enzyme in the tryptophan synthase β-superfamily of pyridoxal phosphate-binding proteins (Glick et al., 2007a,b; Gamalero and Glick, 2015) and is cytoplasmically localized. It has a subunit of mass of ~35–42kD, whereas its natural size is between 100 and 112 kD (Raghuwanshi and Prasad, 2018). This enzyme does not have high affinity for the substrate (1.5–6.0 mM). As a co-factor, pyridoxal phosphate is required for ACC deaminase activity (Glick et al., 2007a,b), and is required for activity of ACC synthase, which catalyzes the synthesis of ACC. Enzyme ACCD exists in the microbial community in very low quantities, and in comparison, to ACC deaminase, ACC oxidase has a substantially higher affinity for ACC (Singh et al., 2015). The level of ethylene in bacterial species depends primarily on activities of ACC oxidase and ACC deaminase (Glick, 2014). Amino acids such as *L*-alanine, *DL*-alanine, and *DL*-valine also stimulate enzyme activity to a modest degree, whereas 4-aminobutanoic acid can stimulate enzyme activity to about the same degree as ACC (Honma, 1983; Raghuwanshi and Prasad, 2018). At pH 8.5, the substrate ACC, as well as the competing inhibitors *L*-alanine and *L*-serine has maximum affinity (Hontzeas et al., 2006; Stress, 2018). The *acdS* genes present in certain bacteria and numerous fungi belonging to different genera are thought to have originated from a collective progenitor (Nascimento et al., 2014). Vertical gene transfer is widespread in many bacteria, while horizontal gene transfer, such as inter-kingdom transfer, also occurs occasionally. The structural genes (*acdS*) and regulatory genes (*acdR*) of ACC deaminase genes have been reported in numerous rhizobacterial groups including endophytic, rhizospheric and root nodulating rhizobia such as *Rhizobium* spp. (Kumar et al., 2016), *Bradyrhizobium* spp. (Greetatorn et al., 2019), *Mesorhizobium* spp. (Senthilkumar et al., 2016) and non-rhizobial groups such as *Burkholderia* spp. (Sarkar et al., 2018a,b), *Pseudomonas* spp. (Azadikhah et al., 2019), *Achromobacter* spp. (Chandra et al., 2020), *Enterobacter* spp. (Kruasuwan and Thamchaipenet, 2018), *Azotobacter* spp. (Viscardi et al., 2016), *Bacillus* spp. (Din et al., 2019), and *Leclercia* spp. (Kang et al., 2019). Regardless, however, even if certain strains of a genus and species possess an *acdS* gene, not all strains of that genus and species have ACCD.

#### Bioinoculation impact of ACC deaminase-producing PGPR: Management of biotic and abiotic stresses

Plants may be exposed to a wide range of environmental stresses, both biotic and biotic. ACCD-containing bacterial species safeguards plants from the deleterious impacts of environmental stresses including drought, salinity, high temperature, waterlogging, excess pesticides, heavy metals, and other xenobiotic contaminants by decreasing the activity of stressor-induced ethylene (Figure 4; Ali and Kim, 2018; Danish et al., 2020; Misra and Chauhan, 2020). The utilization of ACCD-positive PGPR for mitigating multiple abiotic stresses and their positive response on plants appears in Table 2.

**Table 2:** Selected examples of ACCD synthesizing PGPR strains in alleviation of abiotic and biotic stress.

S. No.	ACC deaminase producing PGPR	Source	Used against/host plant	Stress	Application response	References
Salinity stress						
1	*Bacillus mycoides* PM-35	Rhizosphere soil	*Zea mays* (L.)	–	Enhanced chlorophyll, soluble sugar and protein content and capacity to scavenge radical ions	Ali et al. (2022a)
2	*Enterobacter cloacae* ZNP-4	*Ziziphus nummularia*	*T. aestivum* (L.)	–	Increased growth parameters like shoot (41%) and root length (31%), fresh plant weight (28%), dry biomass (29%) and leaf chlorophyll	Singh et al. (2022)
3	*Enterobacter cloacae* PM23	Rhizosphere soil	*Zea mays* (L.)	–	Enhanced the power of radical scavenging, relative water content (RWC), soluble sugars, proteins, phenolic content, total flavonoid content in salt-treated *Z. mays* plants	Ali et al. (2022b)
4	*Bacillus marisflavi* CHR JH 203 and *Bacillus cereus* (BST YS1-42)	Leguminous crop	*Pisum sativum* (L.)	–	Increased dry biomass, biochemical constituents (carbohydrates, protein, reducing soluble sugars, leaf chlorophyll, phenolics and flavonoids)	Gupta et al. (2021)
5	*Glutamicibacter* sp. YD01	Rhizosphere of *Oryza sativa*	*Oryza sativa* (L.)	–	Decreased levels of Na^+^ ion buildup and, electrolyte leakage; improved plant development	Ji et al. (2020)
6	*Bacillus aryabhattai* EWR29	Wheat rhizosphere soil	*T. aestivum* (L.)	–	Mitigated the negative impact of NaCl, significantly enhanced growth, and reduced proline content	Farahat et al. (2020)
7	*Paenibacillus* sp. ACC-06 and *Aneurinibacillusaneurinilyticus* ACC-02	*Allium sativum* (L.) rhizosphere soil	*Phaseolus vulgaris* (L.)	–	Negatively affected NaCl-induced pressure and enhanced biological properties (length, fresh weight, biomass) and photosynthetic capability of plant	Gupta and Pandey (2019)
8	*Serratia grimesii* BXF1	Rhizosphere soil	*Phaseolus vulgaris* (L.)	–	Promoted formation of early root nodules and growth; improved the symbiotic attributes of plants	Tavares et al. (2018)
9	*Bacillus, Acinetobacter* and *Enterobacter*	Soil	*Medicago sativa* (L.)	–	Height, leaf-to-stem ratio, fresh weight, dry biomass, pigments used for photosynthetic energy, nitrogen, phosphorus and potassium content all increased in the plants.	Daur et al. (2018)
10	*Enterobacter* sp.	Soil	*Oryza sativa* (L.)	–	Lowered antioxidative enzymatic responses and NaCl-induced ethylene in bacteria-treated plants; improved plant yield and productivity	Sarkar et al. (2018a,b)
11	*Klebsiella* sp.	Rhizosphere of *T. aestivum*	*Avena sativa* (L.)	–	Reduced salt stress and boosted plant development in salt-stressed soil. Expression profiles of the *rbcL* and *WRKY*1 genes were positively regulated	Sapre et al. (2018a,b)
12	*Pseudomonas* sp., *Bacillus cereus* and *Bacillus* sp.	*Brassica napus* rhizosphere	*Festuca rubra and Brassica napus* (L.)	–	Potentially ameliorated the salinity and enhanced the physiological and biochemical traits of plants	Grobelak et al. (2018)
13	*Bacillus cereus* LB1 and *Bacillus aerius* SB1	Rhizosphere soil	*Carthamus tinctorus*	–	Mitigated toxicity of NaCl and promoted vegetative growth of plant	Hemida and Reyad (2018)
14	*Pseudomonas frederiksbergensis*	Soil	*Capsicum annum* (L.)	–	Increased resistance of plants to NaCl stress observed in bacterial treated plants, as evidenced by increased antioxidant enzymatic activity responsiveness in leaf tissue and lowered hydrogen ion concentrations	Chatterjee et al. (2017)
15	*Bacillus licheniformis* HSW-16	Rhizosphere of *T. aestivum*	*Triticum aestivum* (L.)	–	ACCD-positive PGPR strain positively influenced plant growth by relieving toxic effect of salts	Singh and Jha (2016)
16	*Paenibacilluslentimorbus* B-30488	Rhizosphere soil	*Lycopersicon esculentum*	–	Suppressed growth of phytopathogens and inhibited southern blight disease in tomato; improved overall plant growth	Dixit et al. (2016)
17	*Dietzianatronolimnaea*	Rhizosphere soil	*Triticum aestivum* (L.)	–	Halotolerant PGPR strain increased different antioxidant defensive enzymes and stressor metabolites thus improving salt tolerance ability of plant	Bharti et al. (2016)
18	*Pseudomonas putida*	Desert regions of Rajasthan	*C. arietinum* (L.)	–	Relieved salt-induced toxicity and modulated the growth, physiology, biochemical properties and expression of various stress-related genes	Tiwari et al. (2016)
19	*Variovorax paradoxus* 5C-2	Soil	*Pisum sativum* (L.)	–	Loweredthe proline and MDA content and antioxidant enzymes and enhanced the plant growth	Wang C. et al. (2016), Wang P. et al. (2016), and Wang Q. et al. (2016)
20	*Pseudomonas* sp. ST3	Root nodule of *Vigna unguiculata*	*Vigna unguiculata* (L.)	–	Improved the plant water-relation status, ionic balance, biological attributes, and photosynthetic machinery of peas by relieving the NaCl-induced toxic effect	Trung et al. (2016)
21	*Bacillus* sp., *Zhihengliuellahalotolerans* and *Staphylococcus succinus*	Root nodule of *T. aestivum*	*Triticum estivum* (L.)	–	Improved ion balance, nutritional content and homeostasis	Orhan (2016)
22	*Variovorax paradoxus* 5C-2	Root nodule of *P. sativum*	*P. sativum* (L.)	–	Water uptake, ionic homeostasis, overall growth, dry phyto-mass accumulation, leaf chlorophyll and grain yield of pea plants significantly improved	Wang C. et al. (2016), Wang P. et al. (2016), and Wang Q. et al. (2016)
23	*Pseudomonas stutzeri* A1501	Rhizosphere of *O. sativa*	*Oryza sativa* (L.)	–	Restricted level of salts and improved the development and yield features of plant	Han et al. (2015)
24	*Pseudomonas fluorescens* YsS6	Soil	*Lycopersicum esculentum* (L.)	–	Augmented seedling germination, vigor index (SVI), plant length (root and shoot) and plant dry biomass	Ali et al. (2014)
25	*Bacillus flexus, Isoptericola dokdonensis* and *Arthrobacter soli*	Inner tissues of *Limonium sinense*	*L. sinense* (L.)	–	Protected against salinity effects; increased the flavenoid accumulation	Qin et al. (2014)
26	*Rhizobium leguminosarum*	Pea root nodule	*P. sativum* (L.)	–	Augmented lengths of shoots and roots, dry biomass, chlorophyll synthesis, LHb content and nutrient uptake of plants	Ahmad et al. (2013)
27	*Pseudomonas putida* UW4	Soil	*Lycopersicum esculentum* (L.)	–	Increased expression of mRNA in different ROS-scavenging enzymes and stressor metabolites, i.e., proline	Yan et al. (2013)
Drought stress						
28	*Bacillus megaterium* (MU2)	Maize rhizosphere soil	*T. aestivum* (L.)	–	Potentially increased germination indices, vigor indices (SVI), plant fresh weight and dry biomass	Rashid et al. (2022)
29	*Pseudomonas* sp.	Rhizosphere soil of cereal crop	*Arabidopsis thaliana* (L.)	–	Increased plant survival, LRWC, chlorophyll, glycine betaine, stressor proline, and malondialdehyde content in drought-induced *A. thaliana* plants by 95, 59, 30, 38, 23, and 43%, respectively	Yasmin et al. (2022)
30	*Serratia marcescens* and *Pseudomonas* sp.	Rhizosphere of cereal crops	*T. aestivum* (L.)	–	Both strains potentially improved ROS, water status, osmolyte accumulation, chlorophyll and carotenoids content in plant leaves	Khan and Singh (2021)
31	*Enterobacter cloacae* 2WC2	*Withaniacoagulans* plant	*Zea mays* (L.)	–	Morpho-biological parameters, RWC and antioxidant defence enzymes of PEG-treated plants increased following application of *E. cloacae* strain 2WC2	Maqbool et al. (2021)
32	*Bacillus velezensis* strain D_3_	Rhizosphere soil of rain-fed area		–	Photosynthetic capacity, stomatal conductance, vapor pressure, water-use efficiency, and transpiration rate all improved	Nadeem et al. (2021)
33	*Enterobacter* HS9 and *Bacillus* G9	Soil	*Mucuna pruriens* (L.)	–	Improved water uptake, rate of respiration and synthesis of chlorophyll	Saleem et al. (2018)
34	*Ochrobactrumpseudogrignonense* RJ12, *Pseudomonas* sp. RJ-15 and *Bacillus subtilis* RJ-46	Drought-affected rhizosphere soils	*Vigna mungo* and *P. sativum* (L.)	–	Germination attributes, plant length (root and shoot) and dry biomass enhanced	Saikia et al. (2018)
35	*Mitsuaria* sp. and *Burkholderia*	*Arabidopsis thaliana*	*A. thaliana* and *Zea mays* (L.)	–	Lowered evapotranspiration; altered proline, MDA, and levels of plant hormones.	Huang et al. (2017)
36	*Bacillus pumilus* and *Bacillus firmus*	Rhizosphere of *Solanum tuberosum*	*S. tuberosum* (L.)	–	Enhanced proline content in tubers; greater mRNA expression levels of several ROS scavenging enzymes responsible for increased plant tolerance to salt and drought stress.	Gururani et al. (2013)
37	*Bacillus cereus* AR156, *Bacillus subtilis* SM21 and *Serratia* sp. XY21	Soil	*Cucumis sativus* (L.)	–	Root:shoot ratio and vegetative growth increased	Wang et al. (2012)
38	*Pseudomonas fluorescens* ACC-5	Nodule	*Pisum sativum* (L.)	–	Increased water uptake by plants	Zahir et al. (2008)
39	*Pseudomonas* sp.	Drought-stressed soil	*Pisum sativum* (L.)	–	Increased plant height, leaf-to-stem ratio, fresh plant weight, dry biomass, chlorophyll a, b, and total chlorophyll; increased N, P, and K contents.	Arshad et al. (2008)
Heavy metal stress						
40	*Bacillus gibsonii* (PM11) and *Bacillus xiamenensis* (PM14)	Industrially polluted rhizosphere	*Linumusitatissimum* (L.)	–	Incresed fresh and dry biomass, chlorophyll content, proline concentration, and antioxidant enzymatic activity of plants	Zainab et al. (2020)
41	*Agrobacterium fabrum* and *Leclercia adecarboxylata*	Metal-contaminated rhizosphere	*Zea mays* (L.)	–	Potentially alleviated Cr toxicity and improved the overall growth of plants by reducing metal uptake	Danish et al. (2019)
42	*Rhizobium leguminosarum bv. viciae* 1066S	Metal-contaminated rhizosphere	*Pisum sativum* (L.)	–	Increased shoot biomass, nodulation, nitrogen fixation, water usage efficiency (WUE), and nutritional mineral uptake	Belimov et al. (2019)
43	*Agrobacterium fabrum* (CdtS5) and *Stenotrophomonas maltophilia* (CdtS7)	Cd-contaminated wheat rhizophere	*Tritium estivum* (L.)	Cd	Alleviated Cd toxicity and lowered uptake of Cd; improved growth, chlorophyll content and yield attributes of wheat	Zafar-Ul-Hye et al. (2018)
44	Combination of *Pseudomonas* sp., *Bacillus cereus* and *Bacillus* sp.	Rhizosphere soil	*Festuca rubra* and *Brassica napus* (L.)	Heavy metals	Sequestered the metal, reduced proline, MDA and antioxidant enzymes, reduced metal levels within the plant	Grobelak et al. (2018)
45	*Azotobacter chroococcum*	Metal-contaminated rhizosphere	*Zea mays* (L.)	Heavy metals	Detoxified the metals and increased biological and physiological parameters of the plant	Rizvi and Khan (2018)
46	*Pseudomonas aeruginosa*	Metal-polluted soil	*C. arietinum* (L.)	Heavy metals	Enhanced root length, shoot length, biomass, chlorophyll formation, nodulation, symbiotic attributes and seed yield of plant	Saif and Khan (2018)
47	*Enterobacter aerogenes* MCC 3092	Rhizosphere of *Oryza sativa*	*Oryza sativa* (L.)	Cd	Alleviated phytotoxicity of Cd, reduced level of ethylene, antioxidant enzymes (CAT, SOD, POD), increased growth and chlorophyll content of plants	Pramanik et al. (2018)
48	*Enterobacter ludwigii* (HG 2) and *Klebsiella pneumonia*	*Alternanthera sessilis* and *Cyperus esculentus* rhizosphere	*T. aestivum* (L.)	Cr	Much improved growth promotion of wheat seedlings.	Gontia-Mishra et al. (2016)
49	*Enterobacter* sp., *Serratia* sp. and *Klebsiella* sp.	Rhizospheres of plants growing in mining waste	*Helianthus annuus* (L.)	Pb	Lowered toxicity of Cd, promoted growth features of plants	Carlos et al. (2016)
50	*Pseudomonas fluorescens* and *Bacillus thuringiensis*	Rhizosphere of *Zea mays*	*T. aestivum* (L.)	Cr	Improved plant growth and decreased Cr accumulation in roots and shoots	Shahzadi et al. (2013)
51	*Pseudomonas stutzeri* A1501	–	*Oryza sativa* (L.)	Ni	increased metal tolerance of plants	Han et al. (2015)
52	*Azotobacter* sp.	Metal-contaminated rhizosphere	*Zea mays* (L.)	Pb	Lowered Pb toxicity and enhanced plant biometric parameters, biomass production, chlorophyll *a* and *b* and carotenoids, protein, proline, glutathione *S*-transferase and enzymes of POD and CAT	Hassan et al. (2014)
53	*Ochrobactrum* sp. and *Bacillus* spp.	Slag disposal site	*Oryza sativa* (L.)	Heavy metals	Mitigated toxicity of heavy metals, reduced ethylene level and enhanced overall growth of plants	Pandey et al. (2013)
Organic pollutant stress						
54	*Burkholderia* sp.	Soil	Assorted vegetables	Organic pollutant	Lowered phenol toxicity, thus increasing overall functioning of plants	Chen et al. (2017)
55	*Enterobacter intermedius*, *Bacillus circulans* and *Serratia carnosus*	*Z. mays* and *per nigrum* Rhizosphere soil	*Z. mays* (L.)	Organic pollutant	Improvement in vegetative development of plant was quite noticeable	Ajuzieogu et al. (2015)
56	*Pseudomonas aeruginosa* SLC-2and *Serratia marcescens* BC-3	Contaminated soil	*Avena sativa* (L.)	Organic pollutant	Degraded/detoxified the pollutant and improved biological properties and yield of plants even in petroleum-contaminated soil	Liu et al. (2015)
57	*Acinetobacter* sp.	Ployscyclic aromatic hydrocarbon (PAHs)-contaminated soil	*A. sativa* (L.)	Organic pollutant	DegradedPAHs and hydrocarbons; decreased level of MDA, free proline content and ROS-scavenging enzymes; increased overall performance of plants	Xun et al. (2015)
58	*Pseudomonas aeruginosa* and *Serratia marcescens*	Rhizosphere of Echinochloa	*A. Sativa* (L.)	Organic pollutant	A pronounced increase in *A. sativa* plants	Liu et al. (2015)
Agrochemicals stress						
59	*Burkholderiacepacia*	Cabbage rhizosphere	*C. arietinum* (L.)	Pesticide	Alleviated toxicity of glyphosate; enhanced overall plant growth and performance	Shahid and Khan (2018)
60	*Rhizobium leguminosarum*	Root nodules of pea	*P. sativum* (L.)	Pesticide	Improved length, biomass, symbiotic features, nutrient uptake and seed attributes of plants under kitazin stress	Shahid et al. (2019a,b)
Biotic stress						
61	*Pseudomonas putida*	*Withaniasomnifera* rhizosphere soil	*Papaver somniferum* (L.)	*Peronospora* sp. causing downy mildew disease	Biochemical and physiological (stomatal behavior and rate of transpiration) parameters significantly increased	Barnawal et al. (2017)
62	*Bacillus xiamenensis* PM14	Sugarcane rhizosphere	*Saccharum officinarum* L.	*Colletotrichum falcatum* causing red rot disease	Potentially suppressed symptoms of disease, enhanced plant growth, enhanced production of defensive enzymes and content of proline	Xia et al. (2020)
63	*Pseudomonas* sp. strain S3	rhizospheric soil of turmeric (*Curcuma longa*)	*Solanum lycopersicum* (L.)	*Rhizoctonia solani*	Improved morphological features, photosynthetic attributes and osmolytes in plants	Pandey and Gupta (2020)
64	*Paenibacilluslentimorbus* B-30488	rhizospheric soil of tomato	*Solanum lycopersicum* (L.)	*Scelerotiumrolfsii* causing southern blight diseases	Controlled the disease, increased defense enzymes and improved plant growth attributes	Dixit et al. (2016)
65	*Delftiatsuruhatensis* WGR–UOM–BT1	*Rauwolfia serpentina* Rhizosphere	*Solanum lycopersicum* (L.)	*Fusarium oxysporum*	Protected plant from fungal disease; significantly improved characteristic growth features of tomato	Prasannakumar et al. (2015)

In general, every plant has innate ability to withstand the adverse effects of the environment. However, under such stressed conditions, a number of physio-biochemical cascades activated and deactivated upon sensing the type of stresses. Among them, certain phytohormones play important role in stresses plants (Babalola et al., 2003; Glick et al., 2012). However, a number of microorganisms present either in rhizosphere, phyllosphere or endosphere of the plants play crucial role in the sensing and transducing signal to the plants under stressed conditions in coordinated manner. It is well established that ethylene at lower concentration worked as signaling molecules and regulate several gene expression, transcription and translation lead to overall plant development (Shaharoona et al., 2006; Yim et al., 2012; Bal et al., 2013; Ek-Ramos et al., 2019). In contrast, ethylene at higher concentration causes programme cell death, accelerating abscission, aging, inhibiting root elongation, senescence, leaf and fruit drop, etc. Under such circumstances, ACC deaninase either produced by plant or microorganisms cleave the ACC and lowering down the production of excess amount of ethylene even under stressed condition (Glick et al., 1998). Further, microorganisms synthesizing IAA along with endogenous plant IAA could accelerate the amalgamation of the enzyme ACC synthase translating the compound S-adenosyl methionine to ACC being the immediate precursor of ethylene in higher plants (Glick, 2012). It was revealed that phyllosphere methylobacteria distributed in the rice leaves produce the enzyme ACC deaminase, which control the ethylene concentrations level in the rice plant (Chinnadurai et al., 2009). The beneficial impact of ACCD-positive PGPR in the alleviation of various stresses is briefly discussed in the following sections.

##### Salinity stress

Salinity is a critical environmental stress that strongly influences plant productivity worldwide (Pirasteh-Anosheh et al., 2016; Hussain et al., 2019; Singh S. et al., 2021; Singh U. B. et al., 2021). Among the total global cultivable area, ~20% of area suffer from salinity stress; as a direct result of irrigation, this situation is becoming more serious (Kataria and Verma, 2018; Singh U. B. et al., 2021). Globally, the land area affectted by salinity/sodicity is estimated to be over 800 million hectares (MH) (FAO, 2008; Rengasamy, 2010; Dixit et al., 2015). Salinity affects plant physiology *via* differing mechanisms including disruption of chlorophyll synthesis, increased levels of photorespiration and transpiration, and fluctuation in homeostasis in plant cells (Miller et al., 2010; Sahu et al., 2021). Nutrient imbalance due to salinity stress is another variable that adversely affects plant growth and yield (Singh U. B. et al., 2021). This imbalance interrupts proper uptake and transport of nutrients to growing shoots and that ultimately causes mineral deficiencies in the plant (Panda et al., 2017; Singh U. B. et al., 2021). High levels of salt result in oxidative burst of cellular organelles. Increased production of ROS follows, which damages the plasma membrane and adversely affectscellular metabolism and homeostasis. Salinity causes overproduction of ethylene which increases abscission of leaves and petals, and accelerates organ senescence that ultimately leads to premature death of the plant (Zahir et al., 2009; Singh S. et al., 2021). ACCD-containing PGPR have been used to resolve salinity stress in several crops including vegetables and legumes (Shahid et al., 2021a, 2022a,b,c). These PGPR transform ACC to NH_3_ and α-ketobutyrate, which the plant uses as a source of nitrogen, while also mitigating the deleterious effects of salt stress (Siddikee et al., 2012; Barnawal et al., 2014). Even in rather saline environment, salt-tolerant and ACCD-producing bacteria can thrive, and their beneficial characteristics assist plants in overcoming the impacts of stress (Thijs et al., 2014; Han et al., 2021; Sagar et al., 2022).

Microorganisms that survive and flourish in media containing sodium chloride (NaCl) up to 1–33% are known as halotolerant bacteria (Arora et al., 2017; Kumar M. et al., 2019; Singh et al., 2020b). Substantial literature is available on salt-tolerant ACCD-producing PGPR strains that can safeguard plants against the harmful effects of salt. Wang C. et al. (2016), Wang P. et al. (2016), and Wang Q. et al. (2016) found that the ACCD-synthesizing *V. paradoxus* 5C-2 reduced the negative effects of NaCl in pea by enhancing water relations and ion homeostasis, as well as increasing plant growth, dry biomass, chlorophyll synthesis, and yield when pea was grown in a saline environment. Halotolerant strains of *Enterobacter*, *Bacillus* and *Acinetobacter* containing ACCD genes increased plant height, biomass, leaf-to-stem ratio, leaf relative water content (LRWC), production of leaf chlorophyll and nutrient status of *Medicago sativa* (L.) plants cultivated in salinity-stressed agricultural soil (Daur et al., 2018). The early nodulation process and growth of common beans cultivated under high levels of salt stress have been shown to be stimulated by the endophytic bacterium *Serratia grimesii* BXF-1 (Tavares et al., 2018). In a similar study, Ji et al. (2020) reported that *Glutamicibacter* sp. strain YD-01 tolerated exceedingly high salt levels. When treated as a biological inoculant to *Oryza sativa* (L.), this strain exhibited low levels of Na^+^ buildup and decreased electrolyte leakage (EL) during salt treatment, as well as increased plant productivity. In a similar study, two NaCl-tolerant and ACCD-positive PGPR, *Aneurinibacillusaneurinilyticus* ACC-02 and *Paenibacillus* sp. ACC-06, imparted a positive response to morphological attributes (length and biomass), biochemical features, and yield of salt-treated *Phaseolus vulgaris* (L.) by limiting the negative effects of NaCl (Gupta and Pandey, 2019). Wheat (*Triticum aestivum* L.) plants cultivated in saline-sodic soil treated with fertilizer and ACCD positive strains of *S. succinus*, *Zhihengliuella halotolerans* and *Bacillus* sp., either alone or in combination, grew and yielded better than those cultivated in soil treated solely with NaCl (Orhan, 2016; Singh U. B. et al., 2021).

Sapre et al. (2018a,b) reported that a salt-tolerant and ACCD-producing PGPR strain of *Klebsiella* sp. was inoculated to *Avena sativa* plants treated with varying levels of NaCl. The PGPR strain improved plant development under salt stress and progressively regulated the *rbcL* and *WRKY*1 gene expression profiles.

##### Drought stress

Insufficient availability of water, referred to as drought, unfavorably affects crop productivity. Under drought stress many plants physiological and biochemical effects including reduction in water potential, turgor loss, wilting, stomatal closure, and alteration in structures of membranes and proteins are reported (Kaushal and Wani, 2016). Drought stress is documented to slow plant growth, resulting in lower yields, necessitating the use of drought-resistant plant growth techniques. Several researchers have utilized ACCD-producing and drought-tolerant PGPR strains for ameliorating water stress. ACCD-positive PGPR strains *Ochrobactrumpseudogrignonense* RJ-12, *Pseudomonas* sp. RJ-15 and *B. subtilis* RJ-46 were isolated from drought-stressed rhizosphere soil and utilized as bioinoculants to *Vigna mungo* and *Pisum sativum* cultivated under drought stress. ThePGPR strains increased the germination attributes, morphological features and dry weight accumulation in plants (Saikia et al., 2018). Saleem et al. (2018) reported that two ACCD-containing drought-resistant *Enterobacter* HS-9 and *Bacillus* G-9 strains improved overall growth of *Mucuna pruriens* cultivated in drought-stressed conditions. In another crop-based study, two strains of *Bacillus* (*B. pumilus* and *B. firmus*) were reported to enhance the expression levels of mRNA of several ROS scavenging enzymes, and decreased proline concentration in drought-stressed tubers (Gururani et al., 2013). Additionally, the inoculation of ACCD-producing drought-tolerant PGPR strains of *Burkholderia*and *Mitsuaria* sp. recovered from the rhizosphere of *Arabidopsis thaliana* were reported to lower evapotranspiration rate as well as levels of proline and malondialdehyde. Levels of phytohormones were also altered (Huang et al., 2017).

##### Waterlogging stress

Flooding is a common abiotic stress that impacts a wide range of plants. During flooding, plant roots experience anoxia (lack of oxygen), prompting production of ACC that oxidizes ethylene as it moves within the plant. The secreted ethylene has negative consequences on leaves, such as epinasty (rapid nastic motions), chlorosis, necrosis, and lower fruit output (Paul et al., 2016). To eliminate the epinasty response in plants, ethylene production inhibitors likeCO_2_, cobalt chloride, 7-chloro-4-ethoxycarbonylmethoxy-5-methyl-2,1,3-benzothiadiazole, L-α-(2-aminoethoxyvinyl)-glycine (AVG), silver nitrate, and 1-methylcyclopropene (1-MCP) have been used (Jackson, 2008). In addition to these, ACCD-synthesizing PGPR operate as an ACC sink, and their application reduces ethylene levels significantly, protecting plants from flooding stress (Ali and Kim, 2018). Tolerance against waterlogging stress in rice seedlings was enhanced by ACC deaminase-synthesizing *Streptomyces* sp. GMKU 336. The bacteria reduced levels of ethylene and improved root elongation, biomass production, leaf area and chlorophyll content (Jaemsaeng et al., 2018). Etesami et al. (2014) reported that ACC deaminase-positive endophytic *P. fluorescens* strain REN_1_ significantly elongated rice roots, endophytically colonized plants and promoted development of seedlings under waterlogged conditions. Barnawal et al. (2012) observed that ACC deaminase PGPR strains protected *Ocimum sanctum* (L.) plants against waterlogging. Compared to waterlogged plants without bacterial inoculation, the selected bacteria modulated the negative alterations in stress-induced ethylene production, decreased the lipid peroxidation and proline content, and substantially increased the chlorophyll concentration and foliar nutrient uptake in *O. sanctum* plant. Furthermore, ACCD-containing PGPR strains (*P. putida* ATCC17399/pRK415, *Enterobacter cloacae* UW4 and *E. cloacae* CAL2) enhanced various physiological reactions of *S. lycopersicum* (L.) under flooding stress (Grichko and Glick, 2001).

##### Agrochemical stress

Agrochemicals including pesticides, herbicides and fungicides are among the most significant anthropogeniccompounds that adversely affect microbial physiology (Shahid et al., 2019a,b, 2020), composition and functions (Ataikiru et al., 2019; Shahid et al., 2021b; Shahid and Khan, 2022a,b), soil fertility (Sanchez-Hernandez, 2019) and crop productivity (Shahid et al., 2018a,b; Khan et al., 2020). Stress ethylene production causes the agrochemical to obstruct plant development viaunknown mechanisms. Several beneficial pesticide-tolerant soil microbes (PGPR) are reported which can degrade pesticides (Shahid et al., 2019a,b, 2021c; Shahid and Khan, 2019). In addition, a plentiful ACC deaminase-positive and pesticide-tolerant PGPR has been shown to support legumes grown in degraded or stressed soils (Zaidi et al., 2016; Ahmed et al., 2017; Rizvi et al., 2017; Zaidi et al., 2017). Shahid and Khan (2018) reported that glyphosate-tolerant PGPR strain *Burkholderiacepacia* PSBB1 isolated from the contaminated rhizosphere of *Vicia faba* produced considerable ACC deaminase and alleviated the toxicity of the herbicide, and enhanced overall growth and performance of chickpea plants raised in herbicide-amended soil.

##### Heavy metal stress

Soil pollution by heavy metals has become one of the greatest environmental and agronomic challenges worldwide (Ashraf et al., 2019). Certain heavy metals including Zn, Cu, and Co are used by plants in trace quantities; however, they become toxic at higher concentrations and cause deleterious effects to plant growth and development (Dixit et al., 2015). Roots are primarily responsible for nutritient (including metal) uptake by plants. Stress ethylene is produced in soils having high concentrations of heavy metals, which limits root morphogenesis (Saif et al., 2017). Numerous reports existin the literature regarding utilization of metal-tolerant and ACCD-generating PGPR strains capable of optimizing plant growth under heavy metal-stressed conditions (Płociniczak et al., 2014; Pramanik et al., 2018; Manoj et al., 2020). ACCD-positive PGPR support phytoremediation by increasing the uptake of harmful metals by enlarging/improving root growth under metal stress (Santos et al., 2019). In this regard, several agronomists and microbiologists have isolated metal-tolerant and ACCD-producing PGPR strains from different contaminated sites for use as potent bioinoculants for various crops grown in soils contaminated with heavy metals. For instance, single or co-inoculation of metal-tolerating ACCD-producing PGPR strains such as *Bacillus* sp., *B. cereus* and *Pseudomonas* sp. to *Festuca rubra* and *Brassica napus* plants resulted in substantial increases in plant growth and yield (Grobelak et al., 2018). Pandey et al. (2013) reported that metal-tolerant ACCD-positive PGPR strains of *Ochrobactrum* sp. and *Bacillus* spp., when used with rice plantsgrown in heavy metal-contaminated soils, mitigate the toxic effect of metals, reduced ethylene levels and enhanced overall growth of plants. Similarly, two Cr-tolerant PGPR strains, *Enterobacter ludwigii* and *Klebsiella pneumonia*, significantly reduced the toxicity of Cr and promoted seedling germination, and increased protein and carbohydrate content of wheat plants even in the presence of high concentrations of Cr (Gontia-Mishra et al., 2016). Other PGPR strains like *Pseudomonas fluorescens* and *Bacillus thuringiensis* (Shahzadi et al., 2013), *Achromobacter xylosoxidans* and *Bacillus pumilus* (Chandra et al., 2019), *Enterobacter* sp., *Serratia* sp. and *Klebsiella* sp. (Carlos et al., 2016), and *Enterobacter aerogenes* MCC 3092 (Pramanik et al., 2018) are also reported to alleviate toxic ethylene levels vis-à-vis enhanced growth of crops.

##### Temperature (chilling and heat) stress

Extreme (low or high) temperatures cause substantial losses in yield and productivity of crops (Lesk et al., 2016; Wang C. et al., 2016; Wang P. et al., 2016; Wang Q. et al., 2016). Temperature extremes cause plants to modify many metabolic processes (Yadav, 2010). Temperature changes cause drastic alteration in membrane shape, catalytic characteristics, enzyme performance, and nutrient transport (Subramanian et al., 2016). Low temperatures (between 0 and 15°C) cause yield losses in a variety of tropical and subtropical crops. Cold stress generally slows rate of germination, reduces growth, causes yellowing (chlorosis) of leaves, and reduces tiller formation (Yadav, 2010). Chilling causes lesions on leaf surfaces, discoloration, and rapid senescence in horticultural crops due to reduced chlorophyll production. Chilling, like other environmental stresses, results in production of ethylene which inhibits overall plant development. The use of ACCD-synthesizing bacterial strains in *Vitis vinifera* (L.) and *Solanum lycopersicum* (L.) was reported to alleviate chilling stress (Theocharis et al., 2012; Subramanian et al., 2016). Some cold-tolerant and ACCD-negative PGPR strains, *viz.*, *P. frederiksbergensis*, *Sphingomonasfaeni* and *Flavobacterium* sp. were transformed with a plasmid pRKACCharboring the *acdS* gene from *Pseudomonas putida* UW4. The role of these altered PGPRs that overexpressed the *acdS* gene in alleviating chilling stress in *S. lycopersicum* (L.), *Setariaitalica* (L.) and *Eleusine coracana*was investigated (Subramanian et al., 2015; Srinivasan et al., 2017).

##### Air pollution stress

Sulfur dioxide (SO_2_), ozone (O_3_), nitrogen oxides (NOx), and volatile organic compounds (VOCs) are anthropogenicand naturally-occurring pollutants that impart negative impacts to human health and ecosystems (Sharma et al., 2013). Atmospheric pollutants deleteriously affect plants by inhibiting enzyme systems and metabolic activities (Saxena and Kulshrestha, 2016). The increased synthesis of ethylene in plants in response to air pollutants is well documented, and is thought to be one of the key regulators in plant tolerance to air pollution stress, particularly O_3_ exposure (Rao and Davis, 2001). According to one study, inhibition of the ethylene expressing gene resulted in considerable reduction of O_3_-induced leaf damage in tomato plants (Moeder et al., 2002). As a result, bacteria that produce ACC deaminase have received greater attention as a stress management tool for plants suffering from air pollution.

##### Nutrient deficiency

Excessive application of chemical fertilizers in agriculture is costly, andosols considered a potential source of soil and water pollution (Kumar R. et al., 2019). A variety of beneficial ACC deaminase-synthesizing bacteria are known to boost productivity and efficiency of fertilized crops, either directly or indirectly. At low fertilizer application rates, PGPR ACC deaminase activity may reduce ethylene concentrations in wheat plants exposed to nutritional stress by hydrolyzing ACC to α-ketobutyrate and NH_3_ (Hemissi et al., 2019). The authorsfurther claim that PGPR, which comprise ACCD-generating bacteria, might be used in concert with fertilizers to boost nutrient intake and plant development. Multiple studies have demonstrated the critical role of microbially-synthesized ACC deaminase in promoting plant growth, which allows them to withstand abiotic stress and ultimately create a symbiotic interaction between plants and the native rhizosphere (Tahir et al., 2006).

##### Stress from other organic contaminants

Rapid worldwide industrial development and modernization has resulted in the manufacture and release of significant volumes of hazardous organic pollutants into natural habitats. Polycyclic aromatic hydrocarbons (PAHs), petroleum, and other xenobiotics based on hydrocarbons are known to limit crop productivity (Jajoo, 2017; Li et al., 2019). Most plants are stressed by the presence of organic pollutants in soil, which causes them to produce more ethylene. However, the exact mechanisms of excessive ethylene production remain unknown. Organic contaminants such as refrigerants and organic solvents are reported to be degraded by several bacterial species belonging to different genera. In the presence of organic pollutants, ACCD-producing PGPRs have consistently improved plant development (Xun et al., 2015). PGPR can also aid in plant-mediated remediation (phytoremediation) by bio-transforming harmful substances to innocuous forms. ACCD-producing PGPR is known to play a significant role in elongation of roots and overall plant growth, which explains why host plants are superior at phytoremediating organic chemicals. Phenol-degrading PGPR strain *Burkholderia* sp. isolated from phenol-contaminated soil was reported to reduce the phytotoxicity of phenol and improve growth and biochemical activities in plants (Chen et al., 2017). Similarly, ACCD-producing and petroleum-degrading PGPR strains *S. marcescens* BC-3 and *P. aeruginosa* SLC-2 augmented the growth and physiological properties of *Avena sativa* grown in petroleum-contaminated soil (Liu et al., 2015). An ACCD-producing and PAHs-tolerant soil bacterium *Acinetobacter* sp., when applied to *A. sativa* plants cultivated in hydrocarbon-contaminated soil, decreased the MDA, antioxidant enzymes, and free proline contents of shoot tissues and increased yield, photosynthetic pigments, and protein content of plants (Xun et al., 2015). In, another study, two PGPR-degrading *P. aeruginosa* and *S. marcescens* strains isolated from the rhizosphere of *Echinochloa* promoted the growth of *Ascophyllum sativum* (Liu et al., 2015). Application to polluted soil of *Microbacterium* sp. strain F10a-R containing ACC deaminase enzymes and other multifarious PGP features resulted in elimination of pyrene and phenanthrene, both hazardous PAHs, and boosted wheat growth (Sheng et al., 2009).

### Biotic stress

#### Pathogen attack

Plants often respond to attack/infection of bacterial pathogens, fungal pathogens, viruses, and nematodes by increasing ethylene levels in their tissue (Van Loon et al., 2006). Soil application of potent ACCD-producing PGPR strains may reduce injuries from induced ethylene triggered by numerous pathogenic bacteria such as *Agrobacterium tumefaciens* (Toklikishvili et al., 2010), *Pseudomonas syringae* pv. tomato (Indiragandhi et al., 2008), and *Erwinia* spp. (Wang et al., 2000), and those caused by phytopathogenic fungi such as *Pythium aphanidermatum* (El-Tarabily, 2013), *P. ultimum* (Wang et al., 2000), and *Pyriculariaoryzae* (Amutharaj et al., 2012). The PGPR either directly or indirectly inhibit pathogen development by synthesizing a variety of antimicrobial metabolites (Singh et al., 2016a, 2020a). The efficiency and efficacy of varying species and genera of ACC deaminase-producing PGPR strains have demonstrated a positive effect in the suppression of different diseases caused by phytopathogens (Singh et al., 2016b; Shahid et al., 2017). *Bursaphelenchusxylophilus* is a pathogenic nematode commonly known as pine/wood nematode and is associated with by pine wilt disease. This nematode was suppressed by ACC deaminase-containing *B. subtilis* (Nascimento et al., 2013). In an *in-vitro* study, Al-Shwaiman et al. (2022) reported that multi-stress tolerant and biocontrol agent *Beijerinckiafluminensis* supressed the growth of major fungal phytopathogens (*Aletrnariaalternata*, *Rhizoctonia solani*, *Fusariumoxysporum*, *Ustilaginoidea virens*) by producing defensive extracellular enzymes. Dixit et al. (2016) assessed the plant growth-regulating and biocontrol efficiency of ACC deaminase-producing strain *Paenibacilluslentimorbus* B-30488, which suppresses the growth of fungal pathogens and inhibits southern blight disease in tomatoes. Additionally, ACCD containing *Pseudomonas putida* recovered from *Withaniasomnifera* (L.) rhizosphere soil and applied to*Peronospora* sp. causing downy mildew disease infected *Papaver somniferum* (L.) plants. It was observed that the potential ACCD candidate significantly modulated the biochemical and physiological (stomatal behavior and rate of transpiration) parameters by reducing the incidence of disease in plant (Barnawal et al., 2017; Malviya et al., 2020). Based on these data, inoculation of ACC deaminase-containing bacteria to crops suffering from pathogenic stress can protect the plants effectively. In addition, ACCD-synthesizing PGPR strains lower the quantity of ethylene generated in plants infected with soil-borne and foliar disease (Glick, 2014).

Certain plant growth-promoting microorganisms produce the enzyme ACC deaminase, which indirectly promote plant growth by lowering down the ethylene level in plants (Glick, 1995). Under biotic stressed condition, ACC deaminase transcriptionally regulated differently by several biotic factors (Gontia-Mishra et al., 2014). Few reports indicated that *Methylobacterium* spp. (phytopathogenic in nature) modulate plant growth by inhibiting plant pathogens indirectly. ACCD producting *Methylobacterium* spp. synthesized certain polymer degrading pectinase and cellulase, suggesting that they can indirectly induce systemic resistance during pathogen attack (Chinnadurai et al., 2009; Tsolakidou et al., 2019). Under biotic stressed condition, PGPMs produce ACC deaminase which modulates the level of ethylene by hydrolyzing ACC, a precursor of ethylene, in ammonia and a-ketobutyrate (Babalola et al., 2003; Nascimento et al., 2014). The lower concentration of ethylene induced jasmonate dependent pathways in plants which further modulate synthesis of antioxidative biomolecules which in turn reduce the synthesis of reactive oxygen species and superoxide radicals and protect plants from programme cell death against invasion caused by hemi-biotroph and necrotroph. In contrast, ET dependent pathways lead to PCD in the plants attacked by obligate and biotrophs which restrict colonization and invasion of the pathogen. Some time, elevated ET cause premature leaf and fruit drop in the plants attacked by biotrophs (Etesami et al., 2020).

## Concluding remarks and future prospects

In agricultural systems worldwide, environmentally-benign management approaches are necessary to improve food security in the face of constantly changing agro-climatic conditions. The current review focuses on the interaction and mechanistic action of ACCD-synthesizing rhizobacteria on abiotic and biotic stress tolerance induction. It is well recognized that multiple stress-tolerant ACC deaminase-synthesizing bacterial strains are advantageous over other conventional bacterial strains, and can thrive in sufficient numbers in new and stressful environments to impart favorable impacts to crop plants. Under abiotic- and biotic-stressed situations, powerful PGPR strains enhance crop growth and production. Keeping in mind the many significant environmental hazards encountered in agronomic practices from anthropogenic and natural factors, there is an urgent need for a major paradigm shift in agricultural practices. The costs associated with generating and modifying transgenic plants capable of tolerating biotic and abiotic stresses are substantial. To overcome this problem, focus has shifted to the identification and development of ACCD-containing PGPR formulations that support plants in combatting stressed environmental conditions. The survival of such beneficial PGPR strains under harsh circumstances poses a challenge for their large-scale production, yet the exploitation of a powerful PGPR strain is likely to provide wide-ranging solutions to problems in modern agriculture. Research has demonstrated that ethylene balance is crucial for plant growth and development under abiotic stress conditions, and application of PGPR bacteria may be useful in protecting plants from such stresses. Therefore, rhizobacteria should be screened for ACC deaminase production. Utilizing ACC deaminase-synthesizing bacterial strains as biological inoculants for abiotic stress management could be critical for long-term sustainability of agriculture. Furthermore, uncovering the essential mechanistic action of these PGPR strains will help to expand the applicability of this technology.

## Author contributions

MS and MK conceived and designed the study. MS, MK, US, PS, and HS performed the literature search. MS wrote the first draft of the manuscript. MS and US prepared the figures and artwork. MS, MK, US, RK, RS, and AK edited the manuscript. MS, PS, and AM formatted the reference as per Journal’s style. All authors contributed to the article and approved the submitted version.

## Funding

This work is funded by Network Project on Application of Microorganisms in Agriculture and Allied Sectors (AMAAS), Indian Council of Agricultural Research, New Delhi.

## Conflict of interest

The authors declare that the research was conducted in the absence of any commercial or financial relationships that could be construed as a potential conflict of interest.

The reviewer RK declared a shared affiliation with the authors MS, US, and HS to the handling editor at the time of review.

## Publisher’s note

All claims expressed in this article are solely those of the authors and do not necessarily represent those of their affiliated organizations, or those of the publisher, the editors and the reviewers. Any product that may be evaluated in this article, or claim that may be made by its manufacturer, is not guaranteed or endorsed by the publisher.
